# Body mass index and musculoskeletal pain: is there a connection?

**DOI:** 10.1186/2045-709X-21-15

**Published:** 2013-05-20

**Authors:** David R Seaman

**Affiliations:** 1 National University of Health Sciences, SPC-Health Education Center, 7200 66th St, Pinellas Park, FL 33781, USA

**Keywords:** Back pain, Body mass index, Obesity, Metabolic syndrome, Inflammation

## Abstract

**Background:**

Back pain is one of the most common complaints that patients report to physicians and two-thirds of the population has an elevated body mass index (BMI), indicating they are either overweight or obese. It was once assumed that extra body weight would stress the low back and lead to pain, however, researchers have reported inconsistencies association between body weight and back pain. In contrast, more recent studies do indicate that an elevated BMI is associated with back pain and other musculoskeletal pain syndromes due to the presence of a chronic systemic inflammatory state, suggesting that the relationship between BMI and musculoskeletal pains be considered in more detail.

**Objective:**

To describe how an elevated BMI can be associated with chronic systemic inflammation and pain expression. To outline measurable risk factors for chronic inflammation that can be used in clinical practice and discuss basic treatment considerations.

**Discussion:**

Adiposopathy, or “sick fat” syndrome, is a term that refers to an elevated BMI that is associated with a chronic systemic inflammatory state most commonly referred to as the metabolic syndrome. The best available evidence suggests that the presence of adiposopathy determines if an elevated BMI will contribute to musculoskeletal pain expression. It is not uncommon for physicians to fail to identify the presence of adiposopathy/metabolic syndrome.

**Conclusion:**

Patients with an elevated BMI should be further examined to identify inflammatory factors associated with adiposopathy, such as the metabolic syndrome, which may be promoting back pain and other musculoskeletal pain syndromes.

## Introduction

An elevated BMI, due to an increase in adipose tissue mass, is rapidly becoming the norm of our modern society. In the United States, approximately sixty-five percent of adults aged 20 years or older are either overweight or obese [1]. Despite the associations between obesity and diabetes, heart disease, and other chronic diseases, adiposity or fatness, can be misinterpreted to merely represent an excess calorie storage depot due to overeating, rather than an overactive immune/endocrine organ that can generate chronic systemic inflammation [2].

Historically, studies have suggested a weak association between body weight and low back pain [3], and guidelines for the treatment of low back pain have not included diet and weight loss as recommendations [4,5]. Updating this view appears to be in order as an elevated body mass index is considered a risk factor for low back pain chronicity [6,7], and recent studies have indicated that overweight and obese patients are at a higher risk for musculoskeletal pain expression, as are patients with the metabolic syndrome and type 2 diabetes. For example, overweight and obese individuals are more likely to suffer from tension-type or migraine headache, fibromyalgia, abdominal pain, and chronic widespread pain [8,9]. Studies have implicated an elevated by BMI as a promoter of low back pain [10-12]. Obese subjects with hsCRP levels above 3 mg/dL are more likely to report low back pain, compared to obese subjects with normal levels [13].

Studies have demonstrated that local and widespread musculoskeletal pains are more common in patients with the metabolic syndrome [14-16]. Prevalence of neck pain is higher in patients with metabolic syndrome [17]. Shoulder pain is associated with the metabolic syndrome [18]. Achilles, patella, and elbow tendinopathy are associated with the metabolic syndrome [19-21]. Risk of lumbar disc herniation is increased by the metabolic syndrome [22]. Low back and radiating pain is associated with elevated serum lipids and cardiovascular disease risk factors [23-31]. Osteoarthritis is also promoted by the metabolic syndrome [32-35]. Patients with type 2 diabetes have reduced mobility across all joints tested compared to age/weight matched controls [36], and are more likely to develop lumbar stenosis compared to non-diabetics [37,38]. Type 2 diabetes also increases the risk of expressing disc herniation in both the cervical and lumbar spines [39,40].

The term “adiposopathy” has been proposed to describe a “sick fat” inflammatory state versus an overweight state without inflammation [2,41,42]. In other words, not all patients suffer from chronic inflammation when adipose tissue mass increases. This may be the reason for the previous inconsistent correlations between an elevated BMI and low back pain. The emerging interpretation is that adiposopathy and the related metabolic syndrome leads to pain chronicity because the associated non-resolving systemic inflammation is a pathophysiologic state that promotes nociception in injured/dysfunctional musculoskeletal tissues and prevents healing and pain resolution [10,14,35]. This view is consistent with the evidence that chronic non-resolving inflammation is associated with a diversity of seemingly unrelated chronic diseases, such as low back pain, arthritis, atherosclerosis, cancer, chronic obstructive pulmonary disease, asthma, inflammatory bowel disease, neurodegenerative disease, multiple sclerosis, psoriasis, and rheumatoid arthritis [43-51].

Thus, understanding the nature of chronic inflammation, adiposopathy, and the metabolic syndrome is relevant to understanding and treating low back pain and other conditions commonly seen by chiropractors. The remainder of this commentary will discuss these topics.

### What is chronic systemic inflammation?

Chronic systemic inflammation is a “state” of body chemistry that develops over time, which gradually leads to the expression of chronic disease. While this view of inflammation is not novel [52-65], it has yet to be emphasized in the pages of physiology and pathology texts where inflammation is still typically perceived in the context of local physical injury or infection [66,67]. In the traditional context, inflammation is viewed as a normal response to the acute injury/infection, which leads to tissue healing and the resolution of inflammation. As chronic inflammation is typically not a consideration in physiology texts (66), this can lead to a misunderstanding about the nature of common musculoskeletal diseases, of which osteoarthritis is the best example.

Osteoarthritis (OA) is still characterized as a “non-inflammatory, wear and tear” condition [35,67], despite the evidence over the last several decades indicating that it is a chronic inflammatory condition [35,68-73]. In fact, OA joints are inflamed and express the same inflammatory chemistry as found in atherosclerotic vessels [74,75]. And contrary to what might be expected, chondrocytes participate in cartilage degradation by releasing inflammatory mediators [76,77]. Mounting evidence suggests that as with atherosclerosis, OA is a local manifestation of systemic inflammation [32-35,78]. Interestingly, the metabolic syndrome is associated with the expression of OA [35].

Although difficult to visualize, compared with a an injury or infection, the body responds in a similar fashion to “non-overtly injurious” homeostatic challenges. Reduced sleep, stress, sedentary living, and high glycemic index foods, promotes cellular release of inflammatory mediators, most notably are the pro-inflammatory cytokines such as interleukin-1β (IL-1), interleukin-6 (IL-6), and tumor necrosis factor-*α* (TNF). In other words, immune cells release the same mediators whether there is overt tissue injury or noxious homeostatic challenges, such as inadequate sleep, stress, and a high glycemic meal [62,63,74,79-82].

The clinical expression of the systemic pro-inflammatory state takes time to develop and varies among patients, which makes specific cause-effect relationships difficult to identify. Nonetheless, it is known that chronic inflammation, while nonspecific in terms of symptoms, is the pathophysiological state found in most chronic diseases, such as depression, asthma, atherosclerosis, rheumatoid arthritis, diabetes, osteoporosis, Alzheimer’s disease, cancer, and osteoarthritis [75,83,84].

While the clinical expression of the systemic pro-inflammatory state varies among patients, they commonly report poor self-rated health and a depressed affect [85-91], which are risk factors for pain chronicity [6,7]. This inflammatory “state” of ill-health and depression can be induced in many patients when they are given interferon, which causes immune cells to release pro-inflammatory cytokines [91]. The outcome can be a depressed mood, severe fatigue, lethargy, irritability, emotional lability, social withdrawal, lack of concentration and full-blown major depression in a considerable number of patients, which remits when cytokine therapy is withdrawn [91,92]. The direct administration of IL-1 and TNF in animals acts in a dose-dependent manner to generate symptoms of sickness, depression, and pain [93].

Measurements of circulating levels of high sensitivity C-reactive protein (hsCRP) support the contention that the average patient in the United States is inflamed to moderate or high degrees [56], which may explain why depression, fatigue, poor health, and pain are such common symptoms. Levels of hsCRP of <1, 1 to 3, and >3 mg/L denote lower, moderate, and higher relative risk for future vascular events; however, on a practical basis these values should be interpreted as low, moderate, and high systemic inflammation. With this in mind, the average middle-aged American is moderately inflamed with an hsCRP level at about 1.5 mg/L; however, approximately 25% of the US population is highly inflamed with levels of hsCRP greater than 3 mg/L [56]. The recommended anti-inflammatory behavioral changes to reduce hsCRP include diet, exercise, losing weight, and cessation of smoking [56].

### Adiposopathy and chronic inflammation

Multiple interrelated factors lead to weight gain and the development of obesity, the most commonly articulated is a positive caloric balance coupled with sedentary living [41,42]. Inadequate sleep and stress also promote obesity by increasing palatable food consumption, due to increased release of ghrelin and other hormones [94-100]. Less than 6 hrs of sleep per night can undermine dietary efforts to reduce obesity [101] and less than 6 hours or greater than 9 hours of sleep is associated with increased next day pain [102]. Additionally, inadequate sleep and stress independently promote systemic inflammation [103-105]. Sedentary living is also associated with systemic inflammation, which can be modulated with exercise [106-110]. Lees and Booth have gone so far as to call a lack of exercise the “sedentary death syndrome” [111]. A lack of exercise leads to an increase in BMI [112], poorer self-rated health [113,114], and depression [108,115], each of which is a risk factor for developing chronic low back pain [6,7].

The current American diet consists largely of refined, nutrient-free foods, such as sugar, flour, and refined oils. The current American diet is approximately 20% refined sugar, 20% refined grains, 20% refined oils, 15-20% fatty meat, and 10% dairy by calories [1]. Notice that the average American eats virtually no vegetables and fruit and the vast majority of calories (40%) come from refined sugar and flour. Not well known is that high calorie meals consisting of refined carbohydrates and lipids leads to an immediate postprandial response that manifests as hypercoagulability, sympathetic hyperactivity, endothelial dysfunction and the release of inflammatory mediators, such as cytokines and C-reactive protein [62,63]. Postprandial inflammation can occur before substantial elevations in BMI; however, eating in this fashion eventually leads to adipose tissue expansion.

As adiposity increases, there is a fundamental change in the metabolic activity of adipose tissue. In lean individuals, adipocytes exert an anti-inflammatory function by releasing adiponectin and anti-inflammatory interleukin-10, which are associated with health promotion and body repair [116,117]. Adiponectin supports insulin sensitivity and mitochondrial biogenesis in skeletal muscle and interleukin-10 has analgesic and anti-inflammatory immune modulating properties [118-121].

In contrast, as adipocytes increase in size, which is associated with an increase in BMI, a metabolic shift can occur in adipose tissue, such that a systemic chronic inflammatory state develops in certain patients. Indeed, circulating inflammatory mediators, such as hsCRP, TNF and IL-6, were measured in obese individuals and non-obese controls. In obese individuals, an increase in weight, BMI, waist circumference, hip circumference, and waist-hip ratio was correlated to increased levels of inflammatory mediators [122]. These non-invasive measurements can be readily used in clinical practice to get an impression of a patient’s potential inflammatory or pain status.

There is normally a small population of macrophages in lean adipose tissue and they exist in their “M2” or non-activated state [116,117]. However, as adipocytes grow in size, mast cells, lymphocytes, and macrophages can actively enter adipose tissue [123,124], which leads to the transformation of macrophages from M2 to the “M1” or activated state [116,117]. This combination of immune cells causes adipose tissue to behave as an overactive immune organ that promotes chronic systemic inflammation [2,42,116,117,123-127]. Indeed “adiposopathy” is a state in which adipose tissue immune cells are behaving in a fashion that mimics a bacterial infection and autoimmune disorders [123,124]. Thus, it should not be a surprise that overweight individuals are more likely to suffer from pain, malaise and depression.

### Adiposopathy, the metabolic syndrome, and pain expression

As an individual’s waistline continues to increase in size due to gains in adipose tissue mass, additional health/disease markers can change, such as elevations in fasting blood glucose, fasting triglycerides, and blood pressure, as well as reductions in high density lipoprotein (HDL) cholesterol. These five markers reflect the presence of adiposopathy [42], and comprise risk factors for the metabolic syndrome, of which at least three must be present to apply the diagnosis [128-130]. Table 1 is an example of how predictors of the metabolic syndrome and other pro-inflammatory markers can be followed in the clinical setting.

**Table 1:** Markers of chronic inflammation

**Markers**		**Date**	**Date**	**Date**	**Date**
**Metabolic syndrome**	**Abnormal value**				
1. Fasting blood glucose	≥ 100 mg/dL				
2. Triglycerides	≥ 150 mg/dL				
3. HDL cholesterol	< 50 for women; < 40 men				
4. Blood pressure	≥ 130/85				
5. Waist circumference	> 35” women; > 40” men				
**Pro-inflammatory markers**	**Parameters**				
2-hour postprandial glucose	<140 mg/dl = normal				
	140–199 = prediabetes				
	200+ = diabetes				
Fasting triglycerides	<90 mg/dl predicts controlled postprandial response				
hsCRP in mg/L (marker of chronic inflammation)	<1.0 = normal				
	1.0-3.0 = moderate				
	>3.0 = high				
25(OH)D (vitamin D)	32-100 ng/ml (goal >40 ng)				
Body mass index (BMI)	18.5-24.9 = normal				
	25–29.9 = overweight				
	≥30 = obese				
Waist/hip ratio women (risk factor for diabetes)	<0.80 = low risk				
	0.81-.85 = moderate risk				
	>0.85 = high risk				
Waist/hip ratio men (risk factor for diabetes)	<0.95 = low risk				
	0.96-1.0 = moderate risk				
	>1.0 = high risk				
Lack of sleep	Less than 6 hrs				
Stress	Associated with systemic inflammation				
Sedentary living	Associated with systemic inflammation				
Depression	Associated with systemic inflammation				
Self-rated health	Associated with systemic inflammation				

With metabolic syndrome values in mind, the interpretation of glucose and triglyceride values can be further refined to reflect important postprandial inflammatory responses [62]. Population studies have shown that a fasting glucose as low as 90 mg/dL can be associated with a 2-hour post-prandial glucose of >200 mg/dL, which is diagnostic for type 2 diabetes. A 2 hour post-prandial blood glucose level less than 140 mg/dL suggests normal glucose handling; however, data are emerging suggesting that the ideal value may be less. While a fasting triglyceride 150 mg/dl is the cut off for metabolic syndrome, a fasting triglyceride value 90 mg/dl or less is more predictive of postprandial responses [62].

A reduced level of circulating HDL cholesterol is typically viewed as an atherosclerotic plaque risk factor. However, HDL also plays a key role in role in binding absorbed endotoxin to ensure low basal circulating levels, such that reduced HDL cholesterol levels can promote chronic endotoxemia and systemic inflammation [131]. Furthermore, when HDL is burdened by endotoxin, there are multiple pro-inflammatory atherogenic consequences including a suppression of lecithin: cholesterol acyltransferase activity and cholesterol ester transfer protein mass, and a reduced capacity to efflux cholesterol [132], which impact musculoskeletal pain. As mentioned in the introduction, several studies have found a relationship between serum lipids, cardiovascular disease risk factors, and the expression of low back and radiating pain [23-31]. Vitamin D deficiency is also associated with chronic inflammation [133], the metabolic syndrome [134], and low back pain [135].

Approximately 25% of individuals age 40–49, 35% of those age 50–59, and 45% of those over 60 year of age may have the pro-inflammatory metabolic syndrome [136]. In other words, 25-40% of the adult population is chronically inflamed in the fasted state, which is further augmented by repeated consumption of meals that leads to the acute postprandial inflammatory state that was described earlier. When the metabolic syndrome is identified in a patient, the interpretation should be that the patient has transformed into a state of chronic inflammation as evidenced by a host of inflammatory changes [125,137,138] (see Table 2).

**Table 2:** Pro-inflammatory chemistry of the metabolic syndrome

**Hyperglycemia**	**↑ NF-κB**
Hyperinsulinemia	↑ CRP
Hypertriglyceridemia	↑ TNF
Hyperuricemia	↑ IL-6
↓ HDL	↑ Increased white blood cell count
↓ protein synthesis	↑ plasminogen activator inhibitor
↑ protein catabolism	↑ Fibrinogen
↑ gluconeogenesis	↑ Leptin
↑ serum amyloid A	↑ Resistin
↑ angiotensinogen	↓ adiponectin

The pro-inflammatory metabolic syndrome chemistry is known to promote the expression of multiple diseases including type 2 diabetes, cancer, cardiovascular disease, stroke, hypertension, polycystic ovarian syndrome, nonalcoholic fatty liver disease, gallstones, sleep apnea, acne, myopia, male vertex balding, and a reduced age of menarche [138-141]. With such a diverse expression of disease associated with the metabolic syndrome, it is not a surprise that higher postprandial glycemia is considered to be “a universal mechanism for disease progression” [142].

Despite its obvious pervasiveness and associated health risks, it is not uncommon for primary care physicians to fail to identify the presence of the metabolic syndrome [129]. It is important that practitioners of manual therapy avoid this pitfall. As described earlier, the metabolic syndrome and type 2 diabetes are associated with an increased expression of common painful musculoskeletal conditions seen everyday by manual therapists. Additionally, the presence of the metabolic syndrome is directly related to the expression of depression and poor self-rated health [143-147], which are known risk factors for chronic low back pain [6,7].

Knowledge of the metabolic syndrome-pain relationship may be very important, for without it, manual therapists can be led astray to believe that painful conditions not responding to manual care represent “central sensitization syndromes” because it is often assumed that peripheral system is no longer injured or inflamed if manual care is unsuccessful [148]. In contrast, as stated earlier, the emerging impression is that inflammatory chemistry of the metabolic syndrome becomes superimposed over areas of strain or a previous injury and reduces tissue healing and/or leads to ongoing nociception [10,14,35].

As the pro-inflammatory metabolic syndrome is a predecessor of type 2 diabetes, it should not be a surprise that type 2 diabetes is an inflammatory state. At least as early as 2002, review articles outlined how chronic inflammation is the *cause* of insulin resistance and type 2 diabetes [149]. When studies demonstrate that type 2 diabetics have significantly more complications from lumbar fusion surgery [150], the view should not merely be that this is because they have diabetes and do not heal well. While this is true, the correct view is that diabetes is a non-healing state because it is a chronic inflammatory state.

In addition to the pro-inflammatory chemistry outlined in Table 2, patients with type 2 diabetes also have increased circulating painful pro-inflammatory prostaglandins compared to controls [151]. Not surprisingly, these patients are more likely to experience a host of musculoskeletal pain syndromes. Of interest to note is that patients with type II diabetes are known to have altered proteoglycan metabolism in their intervertbral discs, which may promote weakening of the annular fibers and disc herniation [152]. The implication to consider is that the microanatomy of the musculoskeletal system may change in the presence of chronic inflammatory states, such as adiposopathy, the metabolic syndrome, and type 2 diabetes. Recent tendinopathy research supports this contention.

While a waist girth of over 40 inches for men is a risk factor for the metabolic syndrome, it should not be assumed measurements below 40 inches are not associated with inflammation and adiposopathy. A recent study identified that asymptomatic men aged 40 years and older, with a waist girth of more than 33 inches, had the greatest prevalence of Achilles tendinopathy based on ultrasound examination [153]. An earlier study with elite volleyball players also indicated that a waistline measure above 33 inches was associated with an increased risk of tendon pathology; in this case, patella tendinopathy [20]. While a traditional conclusion would be that mechanical loading would be the cause of tendinopathy in such athletes, the authors provided additional insights:

“Waist girth is a good measure of abdominal adipose tissue, and this tissue releases free fatty acids into the circulation during adipocyte lipolysis, as well as pro-inflammatory cytokines. Free fatty acids and cytokines have been linked to disorders such as heart disease and diabetes. These biochemical substances may also adversely affect tendon function and metabolism and predispose to pathology and abnormal imaging. Therefore, waist girth may have a biochemical as well as a mechanical influence on the development of patellar tendon pathology” [20].

While central accumulation of adipose tissue is known to be harmful to tendons (21), it is unlikely that increased loading adequately explains the development of tendinopathy [19]. It is more likely that tendons are compromised by pro-inflammatory metabolic factors associated with an elevated BMI, such that, “lipid deposition is known to occur in tendons, high cholesterol levels have been observed among individuals with Achilles tendon rupture, and the esterified fraction of cholesterol is elevated in biopsies from Achilles tendinopathy subjects” [19]. In other words, the anatomy, and thus, the integrity of the musculoskeletal system is known to be changed by adiposity, perhaps rendering it more susceptible to injury during mechanical loading. These studies support the suggestion that, “it may be appropriate to redefine our concept of tendinopathy to that of a cardiovascular disease (CVD),” and that “perhaps treating CVD risk factors will improve the treatment of tendinopathy” [19].

### Treatment considerations

While the intent of this commentary is not to provide detailed or specific treatments, as this can vary among patients, general management considerations are appropriate to mention. A growing body of evidence clearly implicates pro-inflammatory body chemistry as an initiator and/or perpetuator of musculoskeletal pain syndromes, and thus, the potential for altered chemistry should be considered during patient assessment, especially when an elevated BMI is identified.

While not all patients with an elevated BMI suffer from a chronic inflammatory state, an elevated BMI should be viewed as a potential initiator/promoter of musculoskeletal pain. The risk factors listed in Table 1 can be used to help determine which patients are systemically inflamed. Some are direct markers, including glucose, triglycerides, HDL cholesterol, hsCRP, and vitamin D, while the remainder are surrogate markers of inflammation.

The treatment approach to reduce adiposopathy and the metabolic syndrome can involve multiple anti-inflammatory lifestyle modifications including dietary changes, nutritional supplements, stress management, exercise, and ensuring adequate sleep. Depending on a practitioner’s training, practice scope, and the potential need for pharmacologic interventions, co-management of certain patients will be likely.

## Conclusion

Historically, an elevated BMI was viewed as a storage depot of excess energy due to overeating and/or a lack of exercise. The systemic pro-inflammatory metabolic consequences of an elevated BMI were unknown until recently, which demands that clinicians modify their views about the influence that weight gain can have on human health.

An elevated BMI may or may not be associated with low back pain and other musculoskeletal pain syndromes. However, as the metabolic syndrome is a universal driver of disease expression, including musculoskeletal pain syndromes, it is incumbent upon the chiropractic profession to identify patients at risk. Identifying adiposopathy and the metabolic syndrome can have a substantial public health impact as patients may only present with musculoskeletal pain and yet have the chronic inflammatory chemistry that promotes heart disease, cancer, and other chronic diseases.

## Competing interests

While Dr. Seaman is a paid consultant for Anabolic Laboratories, a manufacturer and distributer of nutritional supplements, no supplements are discussed in this article and he was not financed to write this article.

## Author contribution

DRS is the sole author of this manuscript.
